# Integrative Omics Analysis of Three Oil Palm Varieties Reveals (Tanzania × Ekona) TE as a Cold-Resistant Variety in Response to Low-Temperature Stress

**DOI:** 10.3390/ijms232314926

**Published:** 2022-11-29

**Authors:** Mumtaz Ali Saand, Jing Li, Yi Wu, Lixia Zhou, Hongxing Cao, Yaodong Yang

**Affiliations:** 1 Hainan Key Laboratory of Tropical Oil Crops Biology/Coconut Research Institute, Chinese Academy of Tropical Agricultural Sciences, Wenchang 571339, China; 2 Department of Botany, Shah Abdul Latif University, Khairpur 66020, Sindh, Pakistan

**Keywords:** oil palm, transcriptome, proteome, TE, low-temperature stress, cold tolerance

## Abstract

Oil palm (*Elaeis guineensis* Jacq.) is an economically important tropical oil crop widely cultivated in tropical zones worldwide. Being a tropical crop, low-temperature stress adversely affects the oil palm. However, integrative leaf transcriptomic and proteomic analyses have not yet been conducted on an oil palm crop under cold stress. In this study, integrative omics transcriptomic and iTRAQ-based proteomic approaches were employed for three oil palm varieties, i.e., B × E (Bamenda × Ekona), O × G (*E. oleifera* × *Elaeis guineensis*), and T × E (Tanzania × Ekona), in response to low-temperature stress. In response to low-temperature stress at (8 °C) for 5 days, a total of 5175 up- and 2941 downregulated DEGs in BE-0_VS_BE-5, and a total of 3468 up- and 2443 downregulated DEGs for OG-0_VS_OG-5, and 3667 up- and 2151 downregulated DEGs for TE-0_VS_TE-5 were identified. iTRAQ-based proteomic analysis showed 349 up- and 657 downregulated DEPs for BE-0_VS_BE-5, 372 up- and 264 downregulated DEPs for OG-0_VS_OG-5, and 500 up- and 321 downregulated DEPs for TE-0_VS_TE-5 compared to control samples treated at 28 °C and 8 °C, respectively. The KEGG pathway correlation of oil palm has shown that the metabolic synthesis and biosynthesis of secondary metabolites pathways were significantly enriched in the transcriptome and proteome of the oil palm varieties. The correlation expression pattern revealed that TE-0_VS_TE-5 is highly expressed and BE-0_VS_BE-5 is suppressed in both the transcriptome and proteome in response to low temperature. Furthermore, numerous transcription factors (TFs) were found that may regulate cold acclimation in three oil palm varieties at low temperatures. Moreover, this study identified proteins involved in stresses (abiotic, biotic, oxidative, and heat shock), photosynthesis, and respiration in iTRAQ-based proteomic analysis of three oil palm varieties. The increased abundance of stress-responsive proteins and decreased abundance of photosynthesis-related proteins suggest that the TE variety may become cold-resistant in response to low-temperature stress. This study may provide a basis for understanding the molecular mechanism for the adaptation of oil palm varieties in response to low-temperature stress in China.

## 1. Introduction

Oil palm (*Elaeis guineensis* Jacq.) is an essential tropical oil plant that is listed as the most efficient oil-producing crop in the world, with an average global yield of 4.17 tons per hectare [1]. Oil palm has been ranked as the number one source of consumed vegetable oil globally in 2021/2022, with approximately 82 million tons of oil palm and palm kernel oil consumed [2]. Oil palm is a perennial monocot belonging to the Arecaceae family and is widely cultivated in Southeast Asian countries followed by some African countries, North American countries, and Brazil [3]. However, for research and commercial purposes, the regional and trial planting of oil palm crops is now underway in South China, including Hainan province [4]. The optimal temperature for the growth of oil palm ranges from 24 to 28 °C [3]. Nevertheless, a temperature below 20 °C could reduce the growth of the oil palm crop [4,5]. However, a low temperature of 12 °C may adversely affect oil palm growth, fruit development, and/or oil production in sub-tropical regions during the winter season [4].

Cold stress is one of the chief abiotic stresses that can adversely affect the physiology and development of the plant, and crop yield [6]. Plants under cold stress may pass through a series of physiological and biochemical alterations at both the cellular and molecular levels [7]. Subsequently, these changes under cold stress alter the plant’s biological processes, such as respiration, photosynthesis, water circulation and oxygen, and antioxidant enzymatic activity [8]. To cope with such unfavorable conditions, the plant usually acclimatizes with numerous strategies, such as the activation of primary metabolites, raising the levels of antioxidants and chaperones, and changes in gene transcription [9,10,11]. Several genes may be involved in the regulation of gene expression and transcriptional regulatory networks in response to cold stress in plants [12,13].

Transcriptomic and proteomic approaches are useful for identifying RNA transcripts and protein expressions, respectively [14]. One proteomic study identified differentially expressed proteins (DEPs) in oil palms during early fruit development [15]. Nevertheless, iTRAQ-based proteomic studies have been employed in oil palm to reveal DEPs involved in oil palm mesocarp and fatty acid biosynthesis via metabolic pathways during fruit development [16,17,18]. Several transcriptomic studies have also been conducted on developing oil palm zygote embryos, fruit, and flower, and basal trunk oil palm tissue inoculated with pathogen [19,20,21,22]. Previously, transcriptomic analysis has revealed the C-repeat binding factor (CBF)-mediated gene expression pattern in oil palms under cold stress [4].

The analysis of transcriptomics and/or proteomics individually may not provide complete information, whereas the integration of both these approaches could provide complementary information about the plant system [23]. Moreover, the integration of transcriptomics and proteomics not only improves our understanding of fruit ripening but also reveals multiple levels of gene expression in crops [24,25]. Furthermore, multiomics, such as transcriptomics and proteomics (iTRAQ) approaches, have revealed that phytohormones are involved in oil palm seed germination under heat stress [26]. Recently, multiomics analysis integrating the metabolome, transcriptome, and proteome has reported new insights into oil palm leaves in response to salinity stress [2].

Although oil palm, being a tropical oil crop, is supposed to be sensitive to cold stress [3,4], low-temperature stress inhibits the growth of oil palm seedlings [27]. Two intraspecific hybrids, B × E (Bamenda × Ekona) and T × E (Tanzania × Ekona), and one interspecific hybrid, O × G (*E. oleifera × Elaeis guineensis*), have been developed through crossing in order to achieve high-yield and stress-resistant varieties [28,29]. Several reports have demonstrated the role of these hybrids in response to abiotic stresses. Tanzania × Ekona (T × E) has been reported as a cold-tolerant variety [29,30]. The progenies of B × E and O × G have exhibited drought tolerance [27,31]. Nonetheless, both B × E and T × E varieties might show tolerance to drought stress conditions [27]. Notably, hybrids from crossed B × E have also been reported as cold- and drought-tolerant [28]. We hypothesized that T × E and/or B × E might enhance cold tolerance in terms of transcriptome and proteome analysis in response to low-temperature stress. Notwithstanding, there is no evidence in the literature of integrative proteome-wide iTRAQ and transcriptomic study of oil palm leaf in response to low temperature. Thus, in this study, we employed an integrative omics approach, including transcriptomics and proteomics based on iTRAQ, to analyze oil palm leaves under cold stress. Subsequently, leaves of three oil palm varieties, i.e., B × E, O × G, and T × E, were kept at a low temperature (8 °C) for five days for transcriptomics and proteomics analysis. We found that T × E may be more tolerant of low-temperature stress than other varieties, based on transcriptomics and iTRAQ-based proteomics analysis.

## 2. Results

### 2.1. Transcriptome and Proteome Analysis for Oil Palm Varieties under Low-Temperature Stress

**Figure 1:**
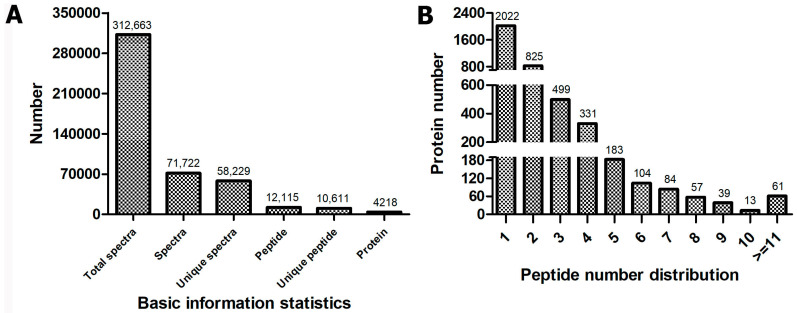
General information of oil palm iTRAQ-based proteomic data output. (**A**) Basic information statistics. (**B**) Peptide number distribution.

For transcriptome identification, a total of six digital gene expression (DGE) libraries were constructed under controlled (28 °C) and low-temperature (8 °C) for three oil palm varieties (BE0/OG0, TE0_VS_ BE5/OG5, and TE5). Each library produced over 12 (twelve) million high-quality clean reads except TE5 (Figure S1). The clean reads were mapped onto a reference genome by 82.99% to 86.18%, of which 68.76% to 75.13% were mapped uniquely, whereas the percentage (%) for multi-position reads ranged from 9.57% to 16.79% (Table S1). However, the iTRAQ-based proteomic experiment generated a total of 312,663 spectra using six samples of oil palm leaves at low-temperature stress (8 °C). A total of 71,722 spectra and a total of 58,229 unique spectra were matched to known spectra and unique spectra. Furthermore, 12,115 peptides, 10,611 unique peptides, and 4218 proteins were detected in the oil palm leaf proteome (Figure 1A). A total of 2022 proteins were determined in unique peptide number distribution, while 2–6 peptides, 7–10 peptides, and >11 peptides were 1942, 193, and 61 in number, respectively (Figure 1B).

### 2.2. Differentially Expressed Gene and Protein (DEG and DEP) Identification and Their Correlation in Three Oil Palm Varieties under Cold Stress

**Figure 2:**
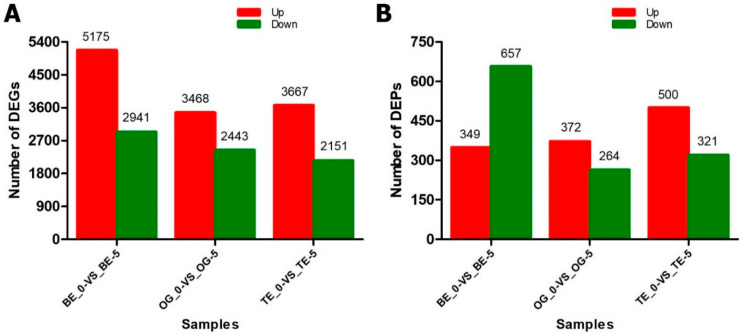
Differentially expressed genes and proteins (DEGs and DEPs) identified in oil palms under cold stress. (**A**) Up- and downregulated genes in three oil palm varieties treated at 8 °C cold stress vs. control at 28 °C for 5 days in leaves. (**B**) DEPs for three oil palm varieties under cold treatment at 8 °C vs. control for five days post-treatment at 28 °C.

In order to identify DEGs and DEPs that were induced by low-temperature treatment (8 °C), the changes in transcription and protein relative abundance levels in leaves of three oil palm varieties were analyzed and calculated by comparing the relative level ratios to controlled treatment at 28 °C. However, comparing the treated and controlled samples for BE-0_VS_BE-5, a total of 5175 up- and 2941 downregulated DEGs were identified (Figure 2A and Table S2), whereas, for OG-0_VS_OG-5, a total of 3468 up- and 2443 downregulated genes and, for TE-0_VS_TE-5, a total of 3667 up- and 2151 downregulated genes were differentially expressed in leaves of OG and TE varieties in response to cold stress (Figure 2A and Tables S3 and S4). Moreover, a total of 2463 differentially expressed proteins were identified in three oil palm leaf samples in response to low-temperature stress. The data comparison was based on fold changes at >1.2 and <0.833 for up- and downregulated proteins, respectively, at *p*-value < 0.05. In BE-0_VS_BE-5, a total of 1006 DEPs were detected, of which 349 and 657 DEPs were up- and downregulated, respectively (Figure 2B and Table S5). For OG-0_VS_OG-5, a total of 636 DEPs were identified, and among the shared DEPs, 372 were upregulated and 264 were downregulated (Figure 2B and Table S6). The TE-0_VS_TE-5 sample shared a total of 821 DEPs, of which 500 were upregulated and 321 were downregulated (Figure 2B and Table S7). The number of upregulated DEGs and DEPs was significantly higher in all the samples except for BE-0_VS_BE-5, in which DEPs were more downregulated compared to DEGs under low-temperature stress (Figure 2B and Tables S5–S7). 

**Figure 3:**
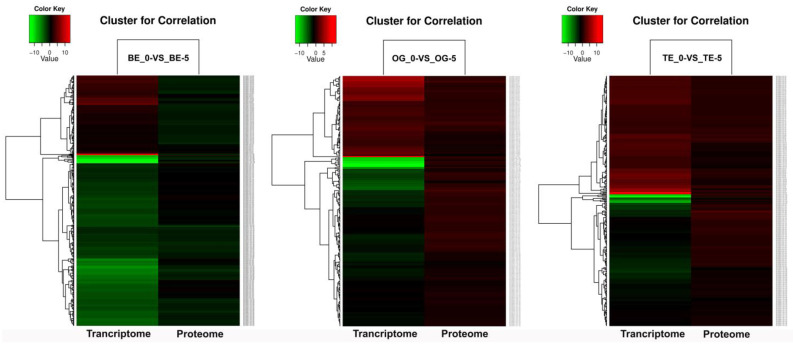
Cluster analysis of DEGs and DEPs for correlation expression of the oil palm transcriptome and proteome under cold stress. Heat map represents the DEGs and DEPs based on the log_2_ relative abundance of the transcriptome and proteome for up- and downregulated (red and green color) genes and proteins of three oil palm varieties.

Additionally, the correlation of the oil palm transcriptome and proteome elucidates the DEGs and DEPs, as shown in Figure 3. The expression pattern clearly shows that a large number of proteins were reduced in the BE-0_VS_BE-5 sample compared to other varieties’ proteomes. Overall, a greater number of DEGs was suppressed in the transcriptome compared to DEPs for the oil palm proteome (Figure 3). These data suggest that the dynamics of protein and/or gene expression regulation may be different in the BE variety in response to cold stress.

### 2.3. GO and Functional Classification and Correlation Analysis of DEGs and DEPs for Three Oil Palm Varieties in Response to Low-Temperature Stress

**Figure 4:**
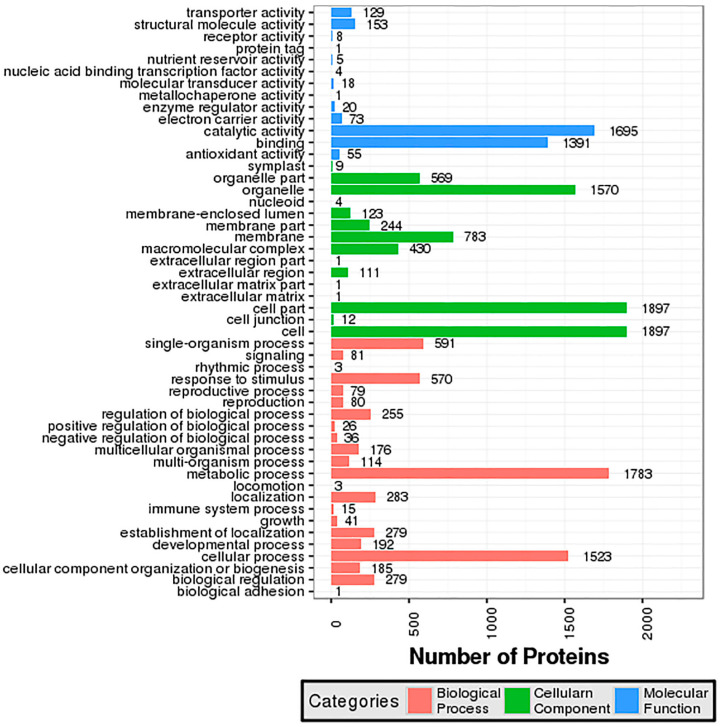
GO analysis for DEPs in oil palm leaves under cold stress. GO categories for identified DEPs based on iTRAQ proteomics in the oil palm proteome are demarcated in three different colors at the bottom of the graph.

GO analysis for DEGs revealed 18, 12, and 11 categories for biological process, cellular component, and molecular function, respectively, in the BE and OG varieties, whereas the TE variety has 16, 12, and 10 categories for biological process, cellular component, and molecular function classes, respectively (Figure S2). On the contrary, the DEPs were enriched into 23, 15, and 13 categories for biological process, cellular component, and molecular function, respectively (Figure 4). Different processes such as metabolic, cellular, single-organism, localization, response to stimulus, biological regulation, and establishment of localization were the top biological processes in most DEGs and DEPs for all three varieties. The DEGs and DEPs for entire varieties were top for enrichment with cell, cell part, organelle, membrane, macromolecular complex, organelle part, and membrane part in GO cellular component analysis (Figure 4 and Figure S2). Additionally, DEGs and DEPs for binding and activity functions such as catalytic, binding, transporter, and structural molecule were found to be the largest groups in molecular function for oil palm varieties under low-temperature stress (Figure 4 and Figure S2).

On the other hand, the correlation of the oil palm transcriptome and proteome also revealed 23, 15, and 13 categories for biological processes, cellular components, and molecular functions, respectively (Figure S3). Several processes, including metabolic (26.44%), cellular (22.68%), single organism (9.05%), response to stimulus (8.8%), biological regulation, and establishment of localization (>4%) were the most enriched in biological processes for the transcriptome and proteome of oil palm. However, cell/cell part (both with 24.92%), organelle (20.51%), membrane (10.33%), organelle part (7.35%), and macromolecular complex (5.28%) were the top categories which enriched GO cellular correlation for the oil palm transcriptome and proteome. For molecular function correlation, catalytic activity (48.13%), binding (39.30%), structural molecule activity (3.98%), and transporter activity (3.7%) were the most enriched categories in the oil palm transcriptome and proteome in response to low-temperature stress (Figure S3).

**Figure 5:**
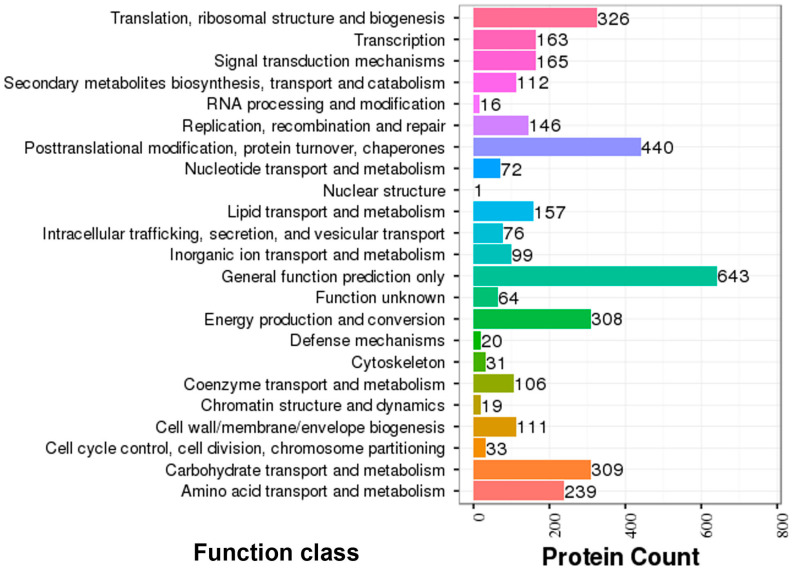
COG functional classification of DEPs identified in oil palm varieties under cold stress. The various colors illustrate each category of functional class.

COG functional classification of DEPs has 23 different classes for the oil palm proteome (Figure 5). General function prediction was the top category, with 643 proteins. Subsequently, post-translational modification, protein turnover, chaperones (440); translation, ribosomal structure, and biogenesis (326); carbohydrate transport and metabolism (309); energy production and conversion (308); amino acid transport and metabolism (239) classes were found to have the greatest protein abundance (Figure 5).

### 2.4. KEGG Pathway Enrichment and Correlation Analysis of Oil Palm DEGs and DEPs in Response to Low-Temperature Stress

**Figure 6:**
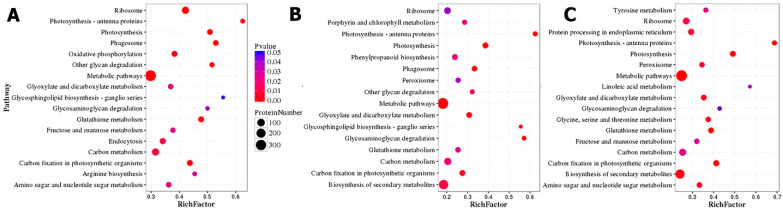
KEGG pathway enrichment analysis of DEPs of oil palm varieties. (**A**–**C**) Top 16/17 in KEGG pathways enrichment of DEPs for three oil palm varieties. RichFactor and P/Q values for each pathway are demarcated at the bottom and right of the graphs, respectively.

KEGG pathway enrichment showed that the metabolic pathway [ko01100] was significantly (p/q < 0.05) enriched in both DEGs and DEPs for the three varieties (Figure 6 and Figure S4). The biosynthesis of the secondary metabolites pathway [ko01110] was the second most enriched in both DEGs and DEPs except for the BE variety’s proteome (Figure 6). The ribosome [ko03010], carbon metabolism [ko01200], photosynthesis [ko00195], carbon fixation in photosynthetic organisms [ko00710], photosynthesis—antenna protein [ko00196], glutathione metabolism [ko00480], and glyoxylate and dicarboxylate metabolism [ko00630] pathways were significantly (*p* < 0.05) enriched in DEPs for three varieties (Figure 6). Notwithstanding, the phagosome [ko04145] pathway was enriched in DEPs for the BE and OG varieties. The peroxisome [ko04146] pathway was enriched in the OG and TE proteome. The amino sugar and nucleotide sugar metabolism [ko00520] and fructose and mannose metabolism [ko00051] pathways were shared by DEPs in the BE and TE varieties. Interestingly, the oxidative phosphorylation pathway was only significantly (*p* < 0.05) enriched in the BE variety’s proteome (Figure 6). 

The plant–pathogen interaction [ko04626], photosynthesis [ko00195], glycolysis/gluconeogenesis [ko00010], circadian rhythm plant [ko04712], carbon fixation in photosynthetic organisms [ko00710], amino sugar and nucleotide sugar metabolism [ko00520], vitamin B6 metabolism [ko00750], endocytosis [ko04144], and regulation of autophagy [ko04140] pathways were enriched in DEGs for three oil palm varieties at (q < 0.06) (Figure S4). The plant hormone signal transduction [ko04075] and nitrogen metabolism [ko00910] pathways were shared by DEGs in the OG and TE varieties at (q < 0.06). The citrate cycle (TCA cycle) [ko00020], glyoxylate and dicarboxylate metabolism [ko00630], and photosynthesis—antenna protein [ko00196] pathways were enriched in DEGs of BE and OG at q < 0.02 (Figure S4). 

**Figure 7:**
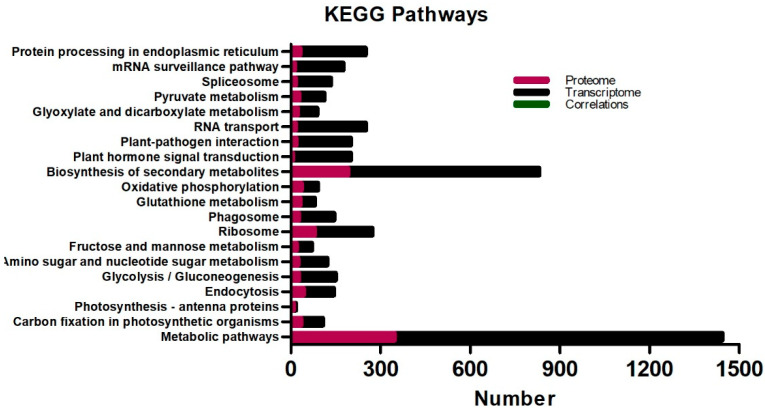
KEGG pathway correlation enrichment analysis of oil palm in response to low-temperature stress. Top 20 in KEGG pathways enrichment involved in DEGs and DEPs of the transcriptome and proteome correlations in oil palm varieties.

Simultaneously, the KEGG pathway correlation of the oil palm transcriptome and proteome found almost similar results to those of individual DEGs and DEPs (Figure 7). The KEGG pathway correlation showed that metabolic pathways [ko01100], the biosynthesis of secondary metabolites pathway [ko01110], ribosome [ko03010], protein processing in the endoplasmic reticulum [ko04141], RNA transport [ko03013], plant–pathogen interaction [ko04626], plant hormone signal transduction [ko04075], and the mRNA surveillance pathway [ko03015] were significantly enriched in the transcriptome and proteome of oil palm varieties (Figure 7). Nonetheless, among the top 20 in KEGG pathway enrichment, the spliceosome [ko03040], glycolysis/gluconeogenesis [ko00010], endocytosis [ko04144], carbon fixation in photosynthetic organisms [ko00710], and other pathways were also enriched in the oil palm transcriptome and proteome correlation in response to low-temperature stress.

### 2.5. Identification of Transcription Factors (TFs) in Three Oil Palm Varieties in Response to Low-Temperature Stress

**Figure 8:**
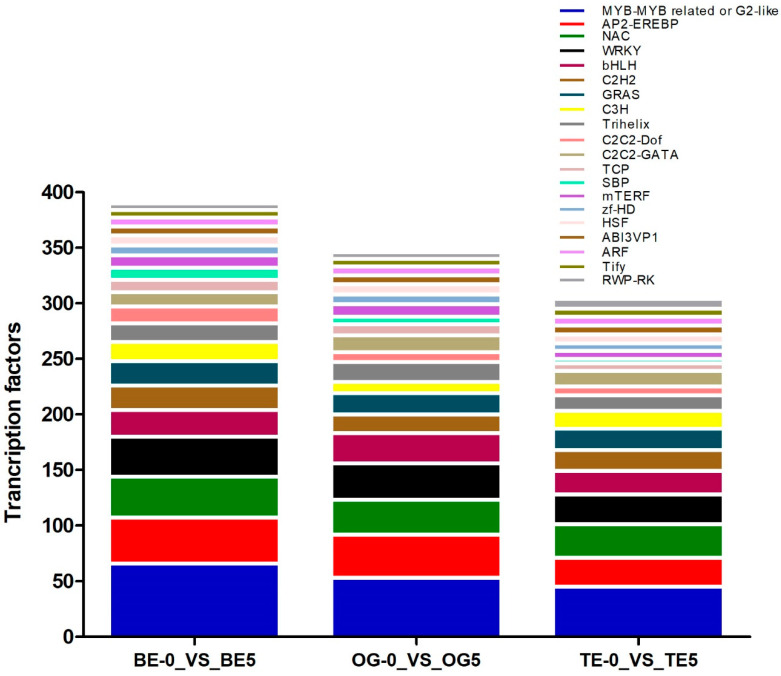
Transcription factors (TFs) identified in oil palm under cold stress. The top 20 TFs involved in the transcriptomes of three oil palm varieties, i.e., BE, OG, and TE.

A total of 540, 458, and 399 TFs were identified in the transcriptomes of the BE-0_VS_BE-5, OG-0_VS_OG-5, and TE-0_VS_TE-5 oil palm varieties, respectively (Table S8). Furthermore, an analysis of the top 20 TFs was conducted as described in Table S9. Among them, MYB-MYB-related or G2-like, AP2-EREBP (ethylene response factor), NAC, and WRKY were found abundantly in the three oil palm varieties (Figure 8). Several other TFs, such as basic helix–loop–helix (bHLH), C2H2, GRAS, C2C2-Dof/GATA, and heat shock transcription factor (Hsf) were also identified in three oil palm varieties (Tables S8 and S9 and Figure 8). This result demonstrates that these TFs genes may play an important role in oil palm transcriptome in response to cold stress. 

### 2.6. Stress-Responsive DEPs in Three Oil Palm Varieties in Response to Low-Temperature Stress

**Figure 9:**
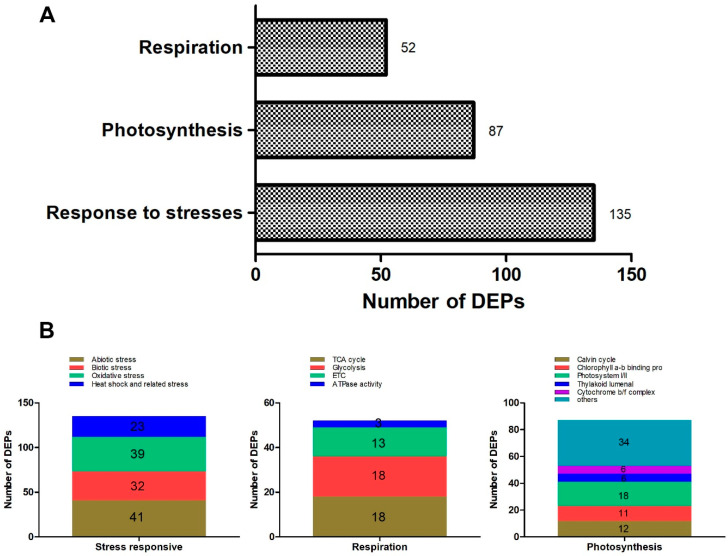
Stress-responsive, photosynthesis, and respiration-related DEPs identified in oil palm proteomes under cold stress. (**A**) Total number of DEPs involved in stress responses, photosynthesis, and respiration in three oil palm varieties. (**B**) Different categories of DEPs for stress responses, photosynthesis, and respiration in the proteomes of three oil palm varieties.

**Figure 10:**
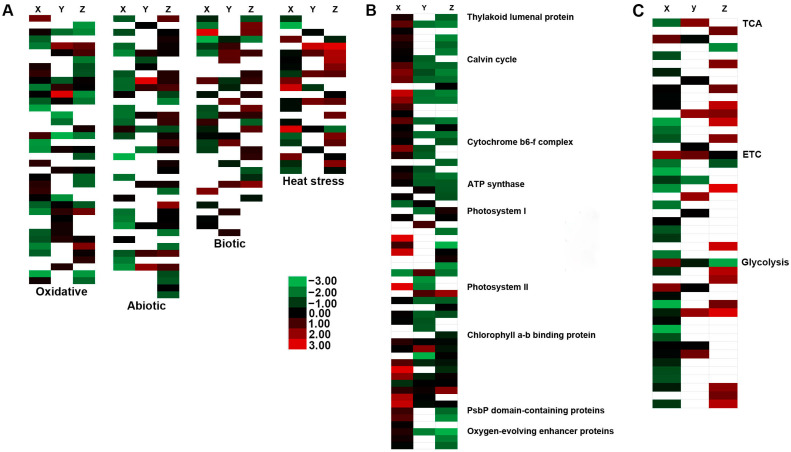
Heatmap illustration for stress response, photosynthesis, and respiration DEPs in oil palm. (**A**) Stress-response DEPs categorized into oxidative, abiotic, biotic, and heat stresses. (**B**) Showing DEPs involved in photosynthesis. (**C**) Displays the DEPs related to respiration for three oil palm varieties. Alphabetical symbols X, Y, and Z denote the BE-0_VS_BE-5, OG-0_VS_OG-5, and TE-0_VS_TE-5 oil palm varieties respectively.

A total of 135 DEPs related to stress were identified in oil palm varieties in response to low-temperature stress (Tables S5–S7 and Figure 9A). In detail, 41, 32, 39, and 23 DEPs were identified for abiotic, biotic, oxidative, and heat shock and related stresses respectively (Table S10 and Figure 9B). Of the total DEPs in oxidative stress, 13, 14, and 12 proteins were upregulated in BE-0_VS_BE-5, OG-0_VS_OG-5, and TE-0_VS_TE-5, respectively (Figure 10A and Table S10). The two superoxide dismutase proteins (SODCP and SOD1) were upregulated in BE-0_VS_BE-5 but were downregulated in the other two varieties, while two superoxide dismutase (SODA and FSD2) proteins were upregulated in OG and TE but not in BE. Peroxidase 4/15/17/72 proteins were significantly downregulated in BE but in the other two varieties were either upregulated or remained undetected. Peroxidase P7 was significantly upregulated in BE but reduced in expression in OG and TE. Glutathione S-transferase F11 (GSTF11) was significantly upregulated in both OG and TE at >1.5-fold changes. Finally, protein DJ-1 homolog D (DJ1D) was significantly upregulated in TE only at expression values <1.9 fold (Tables S7 and S10).

Of the 23 heat shock and related proteins, a greater number of proteins were upregulated in the TE variety (ten), followed by BE (five), and OG (four) (Figure 9B and Figure 10A; Tables S5–S7 and S10). The heat shock proteins (HSP18.6, HSP16.0, HSP70, CLPB1, HSP60 HOP1, and universal stress protein A-like protein) were either reduced or undetected in the three varieties (Table S10). Small heat shock protein (HSP22), heat shock protein binding, heat shock protein 90–5, 20 kDa chaperonin (CPN20), and chaperone protein (ClpC1) were upregulated; however, thirteen heat shock and related proteins were downregulated in the BE variety. Interestingly, these five HSPs were downregulated in the OG and TE varieties (Tables S5–S7 and S10). Additionally, heat shock factor-binding protein 1-like, heat shock protein 83 (HSP83A), heat shock 70 kDa protein, and heat shock 70 kDa (HSP70-17) proteins were upregulated in the OG variety. Intriguingly, these four proteins were downregulated in BE but upregulated in TE in response to low-temperature stress. Only five heat shock and related proteins were downregulated, while ten (10) HSPs were significantly upregulated in the TE variety. At the same time, thirteen (13) and four (4) proteins were downregulated in the BE and OG varieties, respectively. These results indicate that the TE variety is more tolerant and BE seems sensitive to heat shock and related proteins in response to low-temperature stress (Tables S5–S7 and S10).

Of the 32 biotic-responsive proteins, fifteen (15) proteins were downregulated in the BE variety under low-temperature stress (Figure 9B and Figure 10A). Only four proteins, protein phloem protein 2-like A1 (PP2A1), pathogenesis-related protein 1 (PR1), non-specific lipid transfer protein GPI-anchored 1 (LTPG1), and stress-response A/B barrel domain-containing protein HS1, were significantly upregulated in the BE variety (Figure 10A and Tables S5 and S10). The former two proteins were also upregulated in the TE variety. Moreover, in OG, 10 up- and 5 downregulated proteins and, in TE, 12 up- and 6 downregulated proteins were identified in response to low-temperature stress (Tables S6, S7 and S10). Five proteins, disease resistance response protein 206 (PI206), calcium-binding protein CML42 (CML42), glucan endo-1,3-beta-glucosidase, basic vacuolar isoform (HGN1), chlorophyllase-1 (CLH1), and cysteine proteinase inhibitor 12, were significantly upregulated in the OG variety at >1.6 fold changes (Tables S6 and S10). The last protein was also significantly upregulated in the TE variety. Furthermore, ricin, polygalacturonase inhibitor (PGIP), and allene oxide cyclase 3 (AOC3) proteins were upregulated in TE variety at expression values < 1.7 fold (Tables S7 and S10). These data clearly show that the BE variety is more sensitive to biotic stress under low-temperature stress compared to other varieties (see Figure 10A).

In terms of abiotic stress-responsive DEPs, the TE variety has an increased abundance of upregulated proteins compared to the OG and TE varieties (Figure 9B). A total of 19, 9, and 3 proteins were downregulated in BE, TE, and OG, respectively. However, numerous proteins were not detected for the OG variety (Figure 10A; Tables S5–S7 and S10). Only four, namely, calcium-sensing receptor (CAS), 3-ketoacyl-CoA thiolase 2, peroxisomal, (PED1), mitochondrial phosphate carrier protein 3 (MPT3), and zeaxanthin epoxidase, chloroplastic (ZEP-ABA1) proteins were upregulated in the BE oil palm variety in response to low-temperature stress (Table S10). These four proteins were either downregulated or not detected in OG and TE oil palm varieties. Glutamate decarboxylase (GAD1 and GDH2) proteins were upregulated in TE and OG, respectively. ABC transporter C family members (ABCC2 and ABCC14) were upregulated in TE and ABCF4 was downregulated in OG. Aspartic protease in guard cell 1/2 (ASPG1/ASPG2), probable aquaporin PIP1-2 (PIP1-2), lactoylglutathione lyase (GLYI-11), glutathione S-transferase F10 (GSTF10), late embryogenesis abundant protein (LEA14-A), plasma membrane-associated cation-binding protein 1 (PCAP1), and annexin D1/D4 (ANN1/ANN4) proteins were significantly upregulated in the OG and TE varieties. One protein, glutathione S-transferase U17 (GSTU17), was upregulated in the OG variety only. Concurrently, these entire proteins were either significantly downregulated or not detected in the BE variety (Figure 9A and Table S10). Protein embryonic flower 1 (EMF1), probable linoleate 9S-lipoxygenase 5 (LOX1.5), and probable aquaporin (PIP1-2) proteins were significantly upregulated only in the TE variety. Glycine-rich RNA-binding protein 2 (RBG2) and peptide methionine sulfoxide reductase B1 (MSRB1) were significantly downregulated in TE. The linoleate 13S-lipoxygenase 2-1, chloroplastic (LOX2.1) was downregulated in OG. The status of these proteins was either downregulated or remained undetected in the other two varieties (Figure 10A and Table S10). These results also suggest that TE is a more resistant variety against diverse stimuli under low-temperature stress; nevertheless, BE appears to be a sensitive variety under cold stress. 

### 2.7. DEPs Related to Respiration in Three Oil Palm Varieties in Response to Low-Temperature Stress

A total of 52 respiratory DEPs were identified in the oil palm proteome (Figure 9A and Table S11). Most DEPs belong to the TCA cycle, glycolysis, and electron transport chain (ETC) (Figure 9B). The pattern for differentially expressed proteins was the same here, as it was observed in stress-responsive DEPs for three varieties. Numerous proteins (16) were upregulated in TE compared to OG and BE. In addition, a high number of proteins (35) were downregulated in the BE variety (Tables S5–S7 and S11). In total, six and eight proteins were upregulated in the BE and OG varieties, respectively. Only five and ten proteins were downregulated in TE and OG, respectively (Table S11). Numerous DEPs were not detected (denoted as NA in Table S11) in the three varieties. Malate dehydrogenase (TCA), fumarate hydratase 1 mitochondrial (FUM1) (TCA), 6-phosphofructo-2-kinase/fructose-2,6-bisphosphatase (FKFBP) (glycolysis), fructose-bisphosphate aldolase 3 (glycolysis), and cytochrome c oxidase subunit 5b-1 (ETC) proteins were upregulated solely in the BE variety in response to cold stress. Importantly, all these proteins were downregulated in other varieties except the last one, which was upregulated in the OG variety. The two ATP synthase subunit proteins were upregulated in OG with >1.4-fold changes. One cytochrome b-c1 complex subunit Rieske mitochondrial protein was downregulated in OG with <0.6-fold changes. Furthermore, glyceraldehyde-3-phosphate dehydrogenase 2 (GAPC2), triosephosphate isomerase (TPIP1), NADH dehydrogenase [ubiquinone] iron-sulfur protein 8, cytochrome c1-1, heme protein, mitochondrial (CYCL), dihydrolipoyllysine-residue succinyltransferase component of 2-oxoglutarate dehydrogenase complex 1, and phosphoenolpyruvate carboxylase 2 proteins were significantly upregulated in the TE variety at >1.5-fold changes (Table S11), whereas malate dehydrogenase, mitochondrial (MDH) was downregulated in the TE variety at <0.67-fold changes. These results demonstrate that BE is very sensitive to respiratory proteins compared to other varieties under low-temperature stress.

### 2.8. DEPs Related to Photosynthesis in Three Oil Palm Varieties under Low-Temperature Stress

In total, 87 DEPs were involved in photosynthetic proteins in oil palm varieties (Figure 9A). DEPs related to photosystem I/II, Calvin cycle, and chlorophyll a-b binding proteins were dominant, followed by thylakoid luminal and cytochrome b/f complex (Figure 9B). Unlike respiratory and stress-related DEPs, most proteins were upregulated significantly in BE compared to OG and TE in response to low-temperature stress (Tables S5–S7 and S11). As illustrated in Figure 9B, almost all classes of photosynthesis proteins were expressed in BE, while similar proteins were suppressed, except chlorophyll a-b binding proteins, which were expressed in all three varieties (Table S11). All thylakoid luminal, cytochrome b/f complex, oxygen-evolving enhancer, ribulose bisphosphate carboxylase, and PsbP domain-containing proteins were upregulated significantly in the BE variety, but these entire proteins were either downregulated consistently or undetected in both OG and TE varieties in response to low-temperature stress (Figure 10B and Table S11). Four out of seven proteins for the photosystem II reaction center were upregulated in BE. By contrast, photosystem II reaction center protein H (psbH) was upregulated in both OG and TE at >2.00-fold changes, and photosystem II 22 kDa protein (PSBS) was upregulated in the TE variety only, whereas neither (photosystem II) protein was detected in the BE variety. Two ATP synthase subunit alpha and beta chloroplastic proteins were upregulated in BE, while two ATP synthase delta chain (ATPD) and ATP synthase subunit b′, chloroplastic (ATPG) proteins were upregulated in the OG variety. All ATP synthase proteins were downregulated in the TE variety (Figure 10B and Table S11). These data show that BE appears to increase photosynthetic proteins abundantly compared to other varieties in response to cold stress. 

## 3. Discussion

### 3.1. General Features and Correlation of the Transcriptome and Proteome of Three Oil Palm Varieties in Response to Low-Temperature Stress

Cold stress severely affects plants, leading to a decline in plant development, growth, and germination, and causing a substantial reduction in crop yield [32,33,34]. However, critical/chilling temperatures ranging from 7.5 to 12.5 °C may strongly impede the metabolic processes including photosynthesis and respiration in plants [35]. The oil palm is sensitive to low-temperature stress, which may inhibit the growth of oil palm seedlings [3,4,27]. In this study, we applied transcriptomics (RNA-seq) and iTRAQ-based proteomics approaches in three oil palm varieties to investigate the DEGs and DEPs in response to low-temperature stress. Generally, the varieties OG-0_VS_OG-5 and TE-0_VS_TE-5 were found with upregulated DEGs and DEPs (Figure 2A,B). The BE-0_VS_BE-5 variety downregulated DEPs abundantly, but in contrast, the DEGs were more upregulated in the same variety. However, the pattern of correlation expression showed that TE-0_VS_TE-5 is highly expressed and BE-0_VS_BE-5 is suppressed in both the transcriptome and proteome in response to low-temperature stress. The OG-0_VS_OG-5 variety showed an intermediate pattern of expression of both the transcriptome and proteome (Figure 3). These results indicate that TE might be resistant, and BE could be sensitive, to low-temperature stress.

COG classification for the oil palm proteome has manifested a greater number of proteins for general function prediction and posttranslational modification, protein turnover, and chaperones under low-temperature stress (Figure 5). GO enrichment for both DEGs and DEPs was similar and appeared as metabolic and cellular in biological processes, cell and cell part in cellular, and catalytic and binding activity in molecular functions (Figure 4). These results are in agreement with data previously reported for oil palm integrative omics analysis in seed germination [26]. The KEGG pathways for both DEGs and DEPs and their correlation have shown that metabolic synthesis and biosynthesis of the secondary metabolites pathway were the most enriched in the three varieties (Figure 6, Figure 7 and Figure S4). The plant–pathogen interaction [ko04626] and photosynthesis [ko00195] pathways were significantly enriched in DEGs for three oil palm varieties (Figure S4). Recently, transcriptome analysis for oil palm during zygote embryo development has revealed similar results [22].

### 3.2. Transcription Factors Regulation and Increased Abundance of Stress-Responsive Proteins among Three Oil Palm Varieties

Numerous TFs have been playing a vital role in plant development and environmental stress resistance. Various TFs have been involved in cold-responsive gene expression, and the C-repeat binding factors (CBFs) have been identified as being key transcription factors involved in cold acclimation in Arabidopsis [36,37]. The correlation of CBFs with cold-related genes has been observed in oil palm leaves [3]. The study in question identified that CBF1 and CBF3 genes were significantly positively correlated with cold-response genes in oil palms under cold stress conditions [3]. This study identified numerous TFs regulating three oil palm varieties under low temperatures. MYB-MYB-related or G2-like TFs were found abundantly in three varieties (Figure 8 and Table S9). MYB TFs are deeply involved in abiotic stress, including low-temperature stress conditions in plants [38]. Previously, MYB88/124 TFs were found positively regulate cold tolerance in plants [39]. MYB15 has also played a major role in controlling the expression of CBFs in response to low-temperature stress in Arabidopsis [40]. In this study, MYB-MYB-related TFs were found to be more abundant in the BE variety than in the others. Prior to this, MYB-MYB-related TFs were found in the loquat transcriptome under freezing stress [41]. Several AP2/ERF members have demonstrated their potential role in plant development and abiotic stress mitigation in plants [42]. AP2-EREBP (APETALA2/ethylene-responsive element-binding protein) TFs are important and have played a crucial role in loquat fruitlet transcription profiling under freezing stress tolerance [41]. AP2/ERF TFs were also found in winter rapeseed transcriptome under freezing tolerance [43]. AP2-EREBP could be sub-classified into four sub-families, i.e., DREB/CBF, ERF, CBF, and RAP [44], whereas DREB/CBF TFs have demonstrated their indispensable role in low-temperature stress in the tea plant [45]. We have found that BE and OG were more involved in the regulation of AP2-EREBP TFs than that of TE in response to low temperatures (Table S9). These results suggested that the AP2-EREBP TF family has a significant role in cold acclimation in plants, and the same is the case for oil palm varieties. WRKY and NAC are well-known TF families that are involved in the response to low-temperature stress conditions in various plants [46,47]. Several NAC TF genes have been implicated in multiple abiotic stress, including low-temperature responses in plants [48]. The expression of cotton GhNAC8 and 11 were induced by low temperature, although GhNAC9/10 and 13 have not been involved in cold stress tolerance [49]. The NAC TF family members have been involved in cold stress tolerance in crops [50]. WRKY TFs are widely involved in the cold acclimation of plants. The eight WRKY cold-responsive genes encode TFs in the Arabidopsis transcriptome [51]. This study also found a large number of NAC and WRKY TF family members in three oil palm varieties under cold stress. Similar to this study, the NAC and WRKY TFs have been identified in the peanut and rice transcriptomes in response to cold stress [52,53]. Various TF families such as bHLH, C2H2, GRAS, C3H, Trihelix, and HSF have played a significant role in comparative transcriptome analyses in rice and peanut under chilling/cold stress tolerance [52,53]. Importantly, this analysis also found bHLH, C2H2, GRAS, C3H, Trihelix, and HSF in the top 20 TF families in three oil palm varieties. Generally, more TF families have been identified in BE than in other varieties. This result indicates that those transcription factors may demonstrate their importance in response to low-temperature stress in oil palm crops.

Furthermore, several reports have identified stress-responsive DEPs in response to abiotic stresses including low-temperature stress in plants [54,55,56]. This study has identified a total of 135 stress-related DEPs in three oil palm varieties in response to cold stress. A larger number of DEPs were found in response to abiotic stress, i.e., 41 (Figure 9B and Table S10). The expression analysis revealed that TE upregulated more proteins, with 74% of total expressive proteins. In contrast, the BE variety downregulated a larger number of proteins, with 82% of total expressive proteins for abiotic stress. In the OG variety, 59% of DEPs were unidentified, denoted by “NA” in Table S10; however, only 41% of DEPs showed expression, in which 10 proteins were upregulated and 3 proteins were downregulated (Figure 10A; abiotic). ABC transporter C family members 2 and 14 (ABCC2/14) were expressed in the TE variety, while ABCF4 was suppressed solely in OG. Previously, ABCC2 and 14 were expressed in coconut under low-temperature stress [6]. Two drought stress-responsive proteins, aspartic protease in guard cell 1 and 2 (ASPG1/2), were highly expressed in the OG and TE varieties but suppressed in the BE variety. Two water deprivation/desiccation-related proteins, namely, late embryogenesis abundant protein (Lea14-A) and probable aquaporin (PIP1-2), were significantly expressed in TE and suppressed in BE, while the former protein was also expressed in OG (Table S10). The ASPG1 was expressed, whereas ASPG2 and (Lea14-A) were suppressed, in coconut varieties under cold stress [6]. Glutathione S-transferase F10 (GSTF10) and lactoylglutathione lyase (GLYI-11) were highly expressed in OG. These results are in agreement with the previous findings regarding grafted watermelon and coconut seedlings in response to low-temperature stress [6,54]. Four wounding responsive proteins, that is, probable linoleate 9S-lipoxygenase 4/5, linoleate 13S-lipoxygenase 2-1, chloroplastic, (LOX2.1), and glutamate decarboxylase 1, (GAD1) were induced in OG, while one protein, 3-ketoacyl-CoA thiolase 2, peroxisomal (PED1), was expressed in TE only. Annexin D1/3 and 4 (ANN1/3/4) were also expressed in the OG and TE varieties. Nonetheless, annexin 4 (ANN4) increased the expression of pepper leaves against *B. tabaci* [57]. The annexin-like protein RJ4 and annexin D1 increased their expression under salt treatment in *Medicago* [58]. Studies have provided evidence that the annexin proteins may interact with each other in a calcium-dependent manner to regulate responses to abiotic stress [59]. Importantly, calcium-dependent protein kinase 3 (CPK3) and probable calcium-binding protein (CML18) were induced and reduced in OG/TE and BE, respectively (Table S10). These results suggest that annexin proteins may mediate calcium signaling in oil palm varieties in response to cold stress conditions. Collectively, the TE variety has upregulated an abundant number of abiotic stress-related proteins compared to other varieties (Figure 10). This indicates that TE may be a cold-tolerant variety.

Cold stress can induce the overproduction of ROS that may disrupt cellular redox and cause oxidative damage in plant cells [60]. To cope with oxidative stress, plants have developed an effective antioxidant system in response to cold stress [61,62]. However, ROS-enzymatic scavenging systems, including superoxide dismutase (SOD), ascorbate peroxidase (APX), and glutathione S-transferase (GST), have also been developed in plant systems to protect against cellular damage caused by ROS [60]. We found that superoxide dismutase (Cu-Zn), chloroplastic (SODCP), and SOD1 were upregulated in BE, whereas SOD1 was downregulated in both OG and TE varieties. Two superoxide dismutase proteins, i.e., (SODA) and (FSD2), were significantly upregulated in the OG and TE varieties only (Table S10). In previous iTRAQ-based studies, the abundance of Cu/Zn SOD proteins was increased abundantly in *Anabasis aphylla* and maize seedlings in response to cold stress [8,63]. Remarkably, SODCP has previously shown both up- and down-regulation in two coconut varieties under cold stress [6]. Glutathione S-transferase (GST) may reduce oxidative damage caused by ROS scavenging systems [64]. Our results showed that glutathione S-transferase F11 (GSTF11) was upregulated in both OG and TE, while glutathione S-transferase (DHAR3) and (DHAR2) were upregulated in OG only. Glutathione S-transferase DHAR3 and glutathione S-transferase DHAR2-like proteins were upregulated in cantaloupe crops in response to cold stress [65]. Nonetheless, as antioxidant agents, peroxiredoxin (Prx) and thioredoxin play a vital role in redox signaling [61,66]. In this study, thioredoxin-like protein (CDSP32) and Thioredoxin X (TRX-X) were upregulated in the BE and OG varieties, respectively, whereas CDSP32 was downregulated in the TE variety. Thioredoxin-like protein was downregulated in Hainan tall coconut variety and upregulated in cantaloupe under cold stress [6,65]. Peroxiredoxin-2E-2 (PRXIIE-2) and peroxiredoxin Q (PRXQ) were upregulated in BE and peroxiredoxin-2C (PRXIIC) in both the OG and TE varieties (Table S10). These results are in agreement with cantaloupe fruit proteomics analysis under cold stress [65]. In our previous work, peroxiredoxin proteins were downregulated according to coconut leaf proteomics analysis in response to low-temperature stress [6]. In addition, eleven peroxidase protein family members were found most increased in abundance in the OG variety (Table S10). In particular, peroxidases 4 and 17 were suppressed in BE but expressed in both the OG and TE varieties. Peroxidases 4 and 17 were found to be upregulated consistently in coconut seedlings [6]. Overall, entire varieties have shown an equal number of up- and downregulated proteins in oxidative stress (Figure 10A); thus, the data suggest that these varieties may all play a role in ROS homeostasis against oxidative stress in response to cold stress. 

Molecular chaperones such as heat shock proteins (HSPs), chaperonins, and chaperone DnaJ proteins are crucial protective proteins and are involved in various stress responses [67,68]. In our study, a large number of HSPs were downregulated in BE and a greater number of proteins were upregulated in TE compared to other varieties (Figure 10 and Table S10). Of the five expressed proteins, the two HSPs, heat shock protein 90-5, chloroplastic, and 20 kDa chaperonin, chloroplastic (CPN20), were significantly expressed in BE. The 20 kDa chaperonin, chloroplastic (CPN20) was suppressed in both OG and TE. Previously, both heat shock protein 90 and CPN20 proteins were suppressed in coconut seedlings, while HSP90 was decreased in potato tubers in response to low-temperature stress [6,69]. Heat shock factor-binding protein 1-like and heat shock protein 83 (HSP83A) were significantly increased in OG and TE; however, (HSP83A) was suppressed in the BE variety. Moreover, heat shock protein 81-1 (HSP81-1), heat shock cognate 70 kDa protein 2 (HSC-2), and luminal-binding protein 5 (BIP5) were expressed in TE only, whereas these three proteins were either suppressed or undetected in other varieties. The two endoplasmin homolog, molecular chaperone (HSP90) proteins were suppressed in the BE variety, but their expression was increased in the TE variety (Table S10). In previous studies, HSP83A was expressed in coconut [6]. Both HSP83 and 90 were suppressed in potato tubers and coconut seedlings in response to low-temperature stress [6,69]. The HSC-2 was suppressed in coconut but expressed in potato tuber under cold stress [6,69]. By contrast, an abundant number of HSPs were suppressed in the Golden Empress-308 (GE) variety after 12 days of cold treatment in cantaloupe iTRAQ-based proteomic analysis [65]. Commonly, a higher number of HSPs were suppressed in the BE variety (Figure 10A and Table S10), suggesting that BE may be cold-sensitive in response to low-temperature stress.

Two biotic stress-related proteins, i.e., phloem protein 2-like A1 (PP2A1) and cysteine proteinase inhibitor 12, were significantly expressed in BE. The (PP2A1) was also greatly expressed in the TE variety. Ricin, polygalacturonase inhibitor (PGIP), and Allene oxide cyclase 3, chloroplastic (AOC3) were significantly increased in TE but decreased in BE. The ricin was expressed BenDi but suppressed in XiangShui coconut varieties in low-temperature stress [6]. Glucan endo-1,3-beta-glucosidase (A7PQW3) was consistently suppressed in all varieties, while glucan endo-1,3-beta-glucosidase 5 (Q9M088) was expressed in OG but suppressed in the BE variety (Table S10). Non-specific lipid transfer protein GPI-anchored 1, LTPG1 was expressed in BE but significantly suppressed in TE. Pathogenesis-related protein 1 (PR1) was expressed in BE and TE, but PRI and pathogenesis-related protein 2 were suppressed in OG. Glucan endo-1,3-beta-glucosidase (A7PQW3) was expressed significantly but non-specific lipid-transfer and putative pathogenesis proteins were suppressed in coconut seedlings in response to low-temperature stress [6]. Plant–pathogen interaction could stimulate ROS generation, leading to HR of programmed cell death (PCD) [70]. In this study, we found that hypersensitive-induced response protein 1 (HIR1) was suppressed in BE, while hypersensitive-induced response protein 4 (HIR4) was expressed in OG only (Table S10). Previous reports have found that putative hypersensitive-induced proteins were upregulated in banana, *Halogeton glomeratus*, and coconut leaf proteomic analysis under salinity and low-temperature stress [6,71,72]. There is a possibility of synchronizing the response of plants to various stimuli, including abiotic and biotic stresses [73], and oil palm-led proteomic analysis under low-temperature stress could be involved in multi-stress responses such as biotic, abiotic, heat shock, and oxidative stresses. The BE variety decreased the number of biotic proteins (Figure 10A) compared to other varieties and was demonstrated as a cold-sensitive oil palm variety.

### 3.3. Increased Abundance of Photosynthesis Proteins in BE Compared to Other Oil palm Varieties in Response to Low-Temperature Stress

Cold stress may severely affect several aspects of photosynthesis [74]. In this study, we found that the BE variety greatly increased the expression of photosynthesis proteins. Only two proteins, (P31853) and (P49107), were downregulated, but all photosynthesis-related proteins were significantly upregulated, with 96% of total expressive proteins. By contrast, TE and OG both significantly decreased the photosynthesis proteins (Figure 10B and Table S10). All thylakoid luminal proteins were downregulated in TE, but these were highly expressed in the BE variety. Nonetheless, thylakoid luminal 17.4 kDa protein was downregulated in maize seedlings in response to cold [63]. Chlorophyll a-b binding proteins 1, 2, and M9 were suppressed in maize leaf iTRAQ analysis in response to cold stress [63]. In our study, except for chlorophyll a-b binding protein CP26, chloroplastic (LHCB5, which was suppressed in OG only), all chlorophyll a-b binding proteins were highly expressed in all three varieties. Interestingly, chlorophyll a-b binding protein CP26, chloroplastic (LHCB5) was expressed in BenDi but suppressed in XiangShui coconut varieties in response to cold stress [6]. Chlorophyll a-b binding proteins were significantly upregulated at 12 h but downregulated at 24 h post salinity stress in banana leaf iTRAQ analysis [72], although several chlorophyll a-b binding proteins were significantly reduced in coconut seedlings [6]. In the present study, NAD(P)H-quinone oxidoreductase subunit M protein, ATP synthase subunit (alpha, beta, and delta), and cytochrome b6-f complex proteins were altogether suppressed in the TE variety only (Table S10 and Figure 10B). NAD(P)H-quinone oxidoreductase and ATP synthase (delta and B chain) proteins were previously shown to be suppressed in maize under cold stress [63]. Cytochrome b6-f complex proteins were also suppressed in coconut and *Spirulina* in response to low-temperature stress [6,75]. Oxygen-evolving enhancer (OEE) protein is involved in cold acclimation in wheat crops [76]. OEE was upregulated in cold-tolerant alfalfa and winter barley proteomic analysis [77,78]. In the current study, OEE protein 1 was suppressed in OG, whereas OEE 1, 2, and 3 were suppressed in the TE variety only; however, these all were expressed in the BE variety (Table S10). Nevertheless, one study showed that OEE 1 and 2 were suppressed in cold-tolerant Longyou 7, when compared with cold-sensitive Tianyou 4 in *Brassica* leaf iTRAQ under cold stress [79]. Thus, we speculate that severe inhibition of photosynthesis proteins such as NAD(P)H, ATP synthases, cytochrome b6-f complex, OEE, and thylakoid luminal proteins (Figure 10B and Table S10) may reduce the over-energized state of thylakoid membrane, which could lead to photodamage caused by accumulation of ROS in leaves of the TE variety compared to other varieties [63,79]. Notwithstanding, TE has been reported previously as a cold-tolerant variety [29,30]. Hence, the data reveal that TE may be a cold-tolerant variety compared to other oil palm varieties.

### 3.4. Decreased Abundance of Respiratory Proteins in BE Variety under Low-Temperature Stress

The respiratory process as a center of energy metabolism can generate chemical energy, reduce power, and assemble material for the synthesis of several vital organic components in plant systems [79]. We have observed that the BE variety decreased the abundance of respiratory proteins, with 85% of total expressive proteins. On the other hand, the TE variety has an increased abundance of respiration-related proteins, with 76% of total expressive protein (Figure 10C and Table S10). In the citrate cycle (TCA cycle), more proteins were downregulated in the leaves of BE compared with the TE variety. The two-malate dehydrogenase (MDH) proteins P46488 and Q42972 were upregulated in BE; however, these proteins were downregulated in the OG and TE varieties of palm. One MDH protein (O48905) was suppressed in BE and expressed in OG, while one MDH protein (Q08062) was expressed in TE only. In previous studies, the MDH proteins were suppressed in Brassica [79] and upregulated in *S. apetala* under chilling stress [80]. Citrate synthase, mitochondrial (P49298), and isocitrate dehydrogenase [NAD] (Q8LFC0) proteins were suppressed in BE. Nonetheless, isocitrate dehydrogenase [NAD] (Q945K7) was suppressed in OG, and isocitrate dehydrogenase [NADP] (P50218) was expressed in TE only. The isocitrate dehydrogenase [NADP] proteins were suppressed in brassica and coconut leaf proteomic under cold stress [6,79]. Citrate synthase, mitochondrial was expressed in coconut leaf proteomic analysis under cold stress [6]. Two Succinate-CoA ligase [ADP-forming] subunit beta and alpha-2 proteins (Q6K9N6 and Q6DQL1) were suppressed in BE, whereas Q6K9N6 was expressed in the TE variety. Succinate-CoA ligase [ADP-forming] subunit beta was suppressed in brassica leaves in response to cold stress [79]. Another two TCA cycle proteins, phosphoenolpyruvate carboxylase 2 (Q9FLQ4) and P29194, were suppressed in BE but expressed significantly in TE. These results contradict the cold-stressed coconut leaf proteomic analysis [6]. Except for one (cytochrome c oxidase subunit 5b-1 Q9LW15, which was expressed in TE), proteins related to the cytochrome b/c of the electron-transport chain were suppressed consistently in the BE variety. Importantly, cytochrome c1-1, heme protein (CYCL) (P25076) was suppressed in BE but expressed significantly in the TE variety (Figure 10C and Table S10). In previous studies, entire cytochrome b/c complex proteins were suppressed significantly in coconut leaves in response to low-temperature stress [6], but these proteins were expressed in maize leaves in response to chilling stress [63]. 

Furthermore, three respiratory electron transport chain proteins, NADH dehydrogenase [ubiquinone] O49313, Q9FNN5, and Q9FJW4, were reduced in BE only. The O49313 was suppressed in coconut, and several NADH dehydrogenase [ubiquinone] proteins were suppressed in the root proteomic analysis of *Brassica* in response to low-temperature stress [6,81]. Moreover, all proteins involved in glycolysis were significantly reduced in the BE variety except two, namely, fructose-bisphosphate aldolase (FBPA), cytoplasmic isozyme 1 (P46256), and 6-phosphofructo-2-kinase/fructose-2,6-bisphosphatase (Q9MB58). FBPA (P46256) was upregulated in the TE and OG varieties. The FBPA proteins P17784 and Q9ZU52 were increased in TE (Table S10). FBPA proteins were reduced in *Brassica* leaf in response to cold stress [79]. Triosephosphate isomerase (P48495) was expressed in both OG and TE varieties but reduced in BE. Triosephosphate isomerase was previously reduced in coconut and expressed in *S. apetala* under chilling stress [6,80]. The glycolytic proteins, i.e., pyruvate dehydrogenase E1 component subunit alpha/alpha-1 and beat-2 and two pyruvate kinase, cytosolic isozyme (Q42806 and P22200) proteins, were reduced constantly in BE, whereas Q42806 was expressed in the OG variety. Interestingly, all pyruvate kinase, cytosolic isozyme proteins were significantly expressed in coconut leaves in response to cold stress [6]. Additionally, two pyrophosphate–fructose 6-phosphate 1-phosphotransferase subunit alpha/beta (PFP-ALPHA and BETA) and glyceraldehyde-3-phosphate dehydrogenase 2, cytosolic (GAPC2) were expressed in TE. PFP-ALPHA and GAP were significantly increased in coconut and banana proteomic analysis in response to cold and salinity stress, respectively [6,72]. Three proteins belonging to the ATPase activity class, ATP synthase subunit beta, d, and O mitochondrial, were suppressed in BE, while ATP synthase subunit beta and O were expressed in the OG variety (Table S10). In prior proteomic studies, these proteins were expressed in coconut and in A. aphylla in response to cold stress [6,8], *Halogeton glomeratus* under salinity stress [71], and *S. apetala* under chilling stress [80]. Overall, BE has decreased abundance and TE has increased most of the proteins involved in the TCA cycle and glycolytic pathway (Figure 10C). Simultaneously, both varieties have shown the opposite expression pattern for photosynthetic DEPs (Figure 10B), which may explain why TE seems to be a more tolerant variety in response to low-temperature stress.

## 4. Materials and Methods 

### 4.1. Plant Materials and Low-Temperature Stress Treatments

Seedlings of three oil palm hybrids, namely, B × E (Bamenda × Ekona), O × G (*E. oleifera* × *Elaeis guineensis*), and T × E (Tanzania × Ekona), were grown in a nursery at Coconut Research Institute (CRI), Chinese Academy of Tropical Agricultural Science (CATAS), Wenchang, Hainan, China. Approximately 48 seedlings (one-year-old) germinated in the same nursery were selected for cold treatment and control at 28 °C. For the control group, the seedlings of three varieties were placed in a growth chamber at 28 °C for 5 days under similar (light and dark) photoperiod. Meanwhile, for low-temperature treatment, the seedlings of the three varieties were placed at 8 °C for 5 days in a separate chamber. Four individual oil palm seedlings were selected for each treatment, designated as samples BE-0_VS_BE-5, OG-0_VS_OG-5, and TE-0_VS_TE-5, for both proteomics and RNA-seq analyses. The three leaves from each cold-treated seedling at 8 °C (five days post-treatment for three varieties) and control conditions at 28 °C were harvested and frozen with liquid nitrogen, and were kept at −80 °C for RNA-seq and protein extraction. There were three biological replicates for each analysis.

### 4.2. RNA Extraction and RNA-seq Analysis in Oil Palm Varieties

The total RNA for each sample was extracted using Trizol Reagent (Invitrogen, Waltham, MA, USA) according to the manufacturer’s instructions. The purity and concentration of each RNA sample were determined using a ND-2000 spectrophotometer (NanoDrop, Waltham, MA, USA). However, RNA integrity was observed by gel electrophoresis (1.5% agarose). The sequencing libraries were constructed and then sequenced on an Illumina Hiseq 2000 platform at Beijing Genomics Institute (BGI) (Shenzhen, China). In a nutshell, mRNA was enriched and decomposed by oligo (dT) beads and fragmentation buffer, respectively. Subsequently, mRNAs were reverse transcribed into double-stranded cDNA (dscDNA) using random hexamer primers and were synthesized using dNTPs, RNase H, DNA polymerase I, and buffer. Afterward, ligated products were selected for PCR amplification based on agarose gel electrophoresis and finally sequenced on an Illumina Hiseq 2000 platform. After sequencing, clean reads were obtained by removing reads with low-quality and adapters sequences. The Burrows–Wheeler alignment (BWA) tool was used for clean reads mapping to the reference genome, as in Li et al. [82]. Gene expression level was quantified and normalized as fragments per kilobase of transcript sequence per million (FPKM), as in Li et al. [83]. Correlation values and condition specificity were determined and analyzed following the methodology described by Robinson and Oshlack [84] and Yu et al. [85]. Differentially expressed genes (DEGs) among groups were determined using the NOISeq method of Tarazona et al. [86], following default set criteria. Genes with fold change values ≥ 2 (at *p*-value < 0.001) were regarded as DEGs.

### 4.3. Protein Extraction, Trypsin Digestion, and iTRAQ Labelling

iTRAQ-based proteomic analysis for oil palm leaves was carried out at (BGI) Shenzhen, China. The total proteins of each leaf sample were extracted as described previously by Wang et al. [26]. Protein concentration/quantitation and quality were determined with Bradford assay [87] and (SDS-PAGE), respectively. For protein digestion, the 100 μg of protein from each sample was digested with trypsin gold (Promega, Madison, WI, USA) with the ratio of protein:trypsin = 40:1 at 37 °C overnight. Afterward, the peptides were combined with 100 mM (TEAB) and were collected using 8-plex iTRAQ reagent (Applied Biosystems) following the manufacturer’s instructions. Thereafter, the leaf samples were labelled as 113/116 for TE-0_VS_TE-5, 114/117 for BE-0_VS_BE-5, and 115/118 for OG-0_VS_OG-5. Three biological experiments were performed independently. Finally, all labeled peptides were mixed and vacuum-dried following the of manufacturer’s instructions for further identification.

### 4.4. Peptide Fractionation and LC-MS/MS Analysis

The peptides were separated using a Shimadzu LC-20AB HPLC Pump system. Then, digested peptides were reconstituted with a buffer (5% acetonitrile (ACN), 95% H_2_O (water), adjusted pH to 9.8 with ammonia) to 2 mL and loaded onto a column containing 5-μm particles (Phenomenex). Furthermore, peptides were separated at a flow rate of 1 mL/minute with a gradient of 5% buffer B (95% acetonitrile, 5% H_2_O, adjust pH to 9.8 with ammonia) for ten minutes, 5–35% solvent B for 40 min, 35–95% solvent B for 1 min. The system was maintained in 95% buffer B for three minutes and reduced to 5% within one minute before equilibrating with 5% buffer B for ten minutes. The elution was then performed by measuring absorbance at 214 nm, and fractions were collected every one minute. 

Nano-LC–MS/MS analysis was obtained utilizing a nano-LC equipped with a Q-Exactive Mass Spectrometer. Each fraction was resuspended in buffer A (2% ACN and 0.1% FA in water) and centrifuged at 20,000× *g* for 10 min. The supernatant was loaded onto a C18 trap column 5 μL/min for 8min using a LC-20AD nano-HPLC instrument (Shimadzu, Kyoto, Japan) by the autosampler. Then, the peptides were eluted from the trap column and separated by an analytical C18 column (inner diameter 75 μm). The gradient was run at 300 nL/min increasing from 8 to 35% of buffer B (2% H_2_O and 0.1% FA in ACN) in 35 min, then to 60% in 5 min, then at 80% B for 5 min, and finally returned to 5% in 0.1 min and equilibrated for 10 min. Data acquisition was monitored with a TripleTOF 5600 System (SCIEX, Framingham, MA, USA) equipped with Nanospray III source and controlled with the software Analyst 1.6. Data were acquired with the following MS conditions: curtain gas of 30 psi, ion spray voltage 2.3 kV, atomizing air pressure for 5 psi, and interface heater temperature of 150 °C. High sensitivity mode was used for the data acquisition in its entirety. The accumulation time for MS1 was 250 ms and MS spectra ranged from 350 to 1500 *m/z*. Based on intensity, the 30 product ion scans were collected at a threshold of 120 counts per second with charge-state (2+ to 5+) and dynamic exclusion was set for 1/2 of peak width (12 s). However, with regard to data acquisition for iTRAQ, the collision energy was adjusted to all precursor ions for collision-induced dissociation and the Q2 transmission window for 100Da was 100%.

### 4.5. Identification and Quantification of Proteins

The iTRAQ protein identification process was conducted using the Mascot search engine (Matrix Science, London, UK; version 2.3.02). For protein identification, the mass tolerance was 20 ppm for intact peptide masses and 0.05 Da for fragmented ions, and enzyme specificity was set to trypsin with one missed cleavage. Carbamidomethyl (C), iTRAQ8plex (N-term), and iTRAQ8plex (K) were fixed modifications and oxidation (M) and iTRAQ8plex (Y) were variable modifications. MS/MS data were searched from the representative genome: *Elaeis guineensis* (assembly EG5) using BLAST search at NCBI (https://www.ncbi.nlm.nih.gov/protein/?term=txid51953 (accessed on 24 June 2020)) with 34,435 sequences. The protein that contained at least one unique peptide was set as necessary for the identified proteins. In order to control the rate of false-positives at the protein level, the false discovery rate (FDR) was set at less than 1%, with confidence intervals higher than 95% [43]. However, sequences of positively identified proteins were further carried out for BLAST searching of the UniProt database. Ratios for relative protein quantifications were normalized based on the median average protein quantification ratio. Proteins with fold change values ≥ 1.2 and ≤0.8 at *p*-values ≤ 0.05 were considered as significantly differentially expressed proteins [54].

### 4.6. Bioinformatics Analysis

The functional annotation of DEPs was performed using the Blast2GO program against the non-redundant protein (NR; NCBI) database. Additionally, the functional information was also performed and confirmed using the UniProt (https://www.uniprot.org/ (accessed on 20 August 2020)) database. The COG database (http://www.ncbi.nlm.nih.gov/COG/ (accessed on 20 August 2020)) was used for the classification of DEPs. For gene ontology (GO) classification, both DEGs and DEPs were mapped to GO terms (http://www.geneontology.org/ (accessed on 20 August 2020)). A Kyoto Encyclopedia of Genes and Genomes (KEGG) analysis was performed at (http://www.genome.jp/kegg/ (accessed on 20 August 2020)) at *p*-value ≤ 0.05 as a threshold for GO and KEGG pathway enrichment significance with DEGs and DEPs.

## 5. Conclusions

Integrated omics, based on transcriptomics and proteomics, was applied in three oil palm varieties to analyze DEGs and DEPs in response to low-temperature stress. The KEGG pathways and their correlation for the transcriptome and proteome showed that the major proteins and genes were involved in the metabolic processes and biosynthesis of secondary metabolite pathways in the three oil palm varieties under low-temperature stress. The correlation expression pattern revealed that TE is highly expressed, and BE is suppressed in both the transcriptome and proteome in response to low-temperature stress. The iTRAQ-based proteomics revealed that TE has an increased abundance of stress response and downregulated photosynthesis proteins under cold stress. The accumulation of abiotic, biotic, heat, and oxidative stress-related proteins and severe inhibition of photosynthesis proteins in TE may lead to enhanced ROS scavenging capacity during oxidative stress, and this may explain cold tolerance in the TE variety. Nonetheless, further integration of omics approaches such as metabolomics, ionomics, and phenomics could dissect the biological interaction and molecular mechanism underlying the cold-sensitive and/or cold-tolerant varieties of oil palm crops.

## Data Availability

Not applicable.

## Supplementary Materials

The following supporting information can be downloaded at: https://www.mdpi.com/article/10.3390/ijms232314926/s1.
