# Supplementary material for: Integrative Omics Analysis of Three Oil Palm Varieties Reveals (Tanzania × Ekona) TE as a Cold-Resistant Variety in Response to Low-Temperature Stress
Source: Int J Mol Sci. 2022 Nov 29;23(23):14926. doi: 10.3390/ijms232314926 (PMC9740226; doi:10.3390/ijms232314926)
Supplement: Supplementary file 1 [file ijms-23-14926-s001.zip › Figure S4_.KEGG pathway enrichmnet for DEGs of three oil palm varieties.pdf]

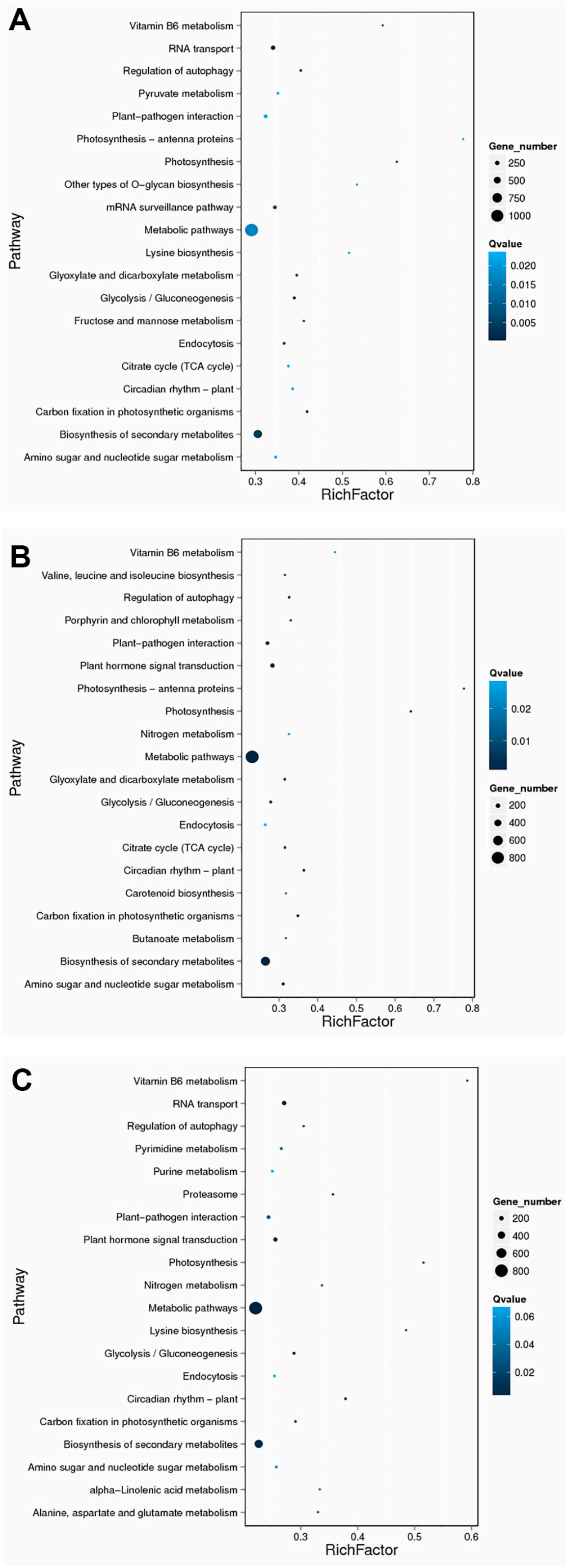

Figure S4. KEGG pathway enrichment for DEGs of oil palm varieties under cold stress. A, B and C represent the BE-0_VS_BE-5, OG-0_VS_OG-5 and TE-0_VS_TE-5 varieties respectively.
