# Supplementary figures and images for: Integrative Omics Analysis of Three Oil Palm Varieties Reveals (Tanzania × Ekona) TE as a Cold-Resistant Variety in Response to Low-Temperature Stress
Source: Int J Mol Sci. 2022 Nov 29;23(23):14926. doi: 10.3390/ijms232314926 (PMC9740226; doi:10.3390/ijms232314926)

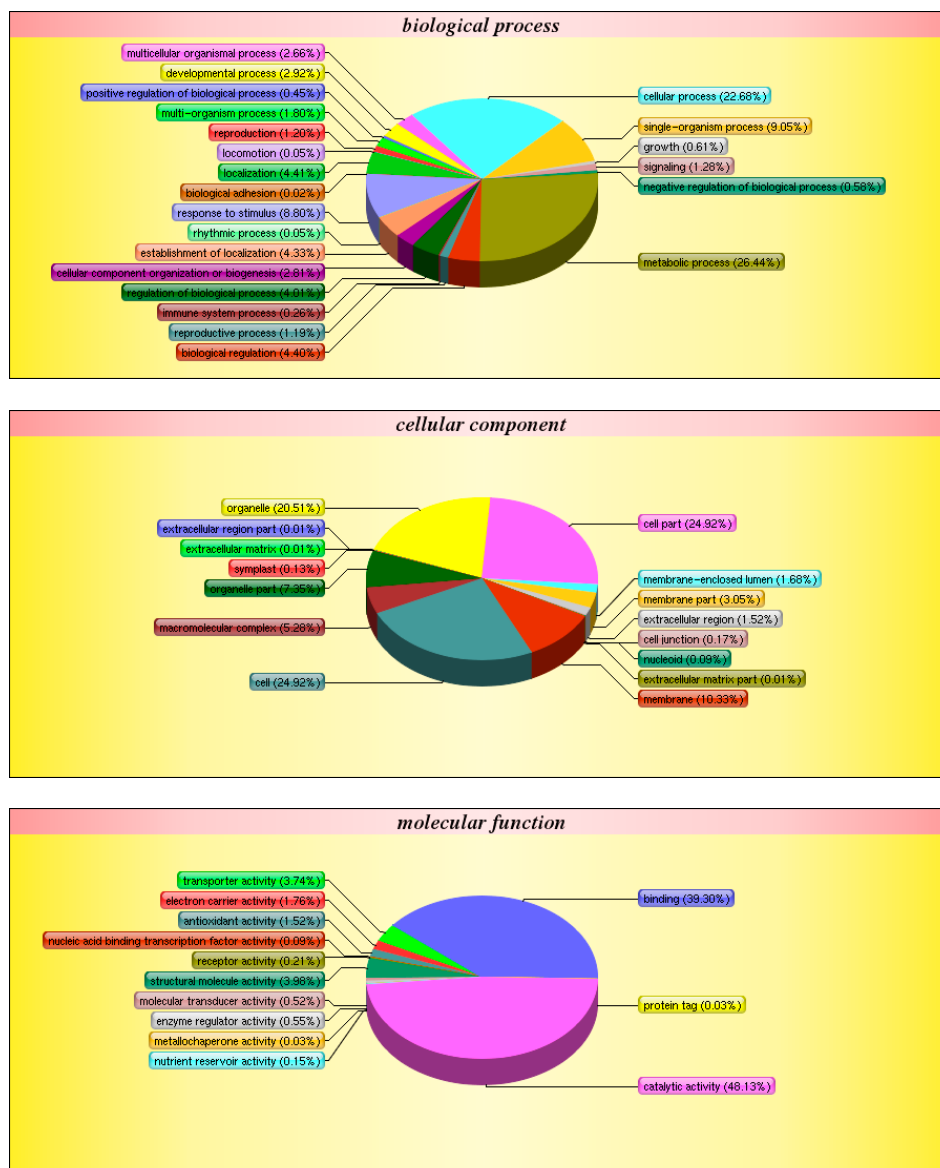

Figure S3. GO correlation for Oil palm Transcriptome and Proteome.

Supplement: Supplementary file 1 [file ijms-23-14926-s001.zip › Figure S3. GO_correlation for oil palm transcriptome and proteome in repsonse to low temperature stress.pdf]
